# Ontogeny of the B- and T-cell response in a primary Zika virus infection of a dengue-naïve individual during the 2016 outbreak in Miami, FL

**DOI:** 10.1371/journal.pntd.0006000

**Published:** 2017-12-21

**Authors:** Michael J. Ricciardi, Diogo M. Magnani, Alba Grifoni, Young-Chan Kwon, Martin J. Gutman, Nathan D. Grubaugh, Karthik Gangavarapu, Mark Sharkey, Cassia G. T. Silveira, Varian K. Bailey, Núria Pedreño-Lopez, Lucas Gonzalez-Nieto, Helen S. Maxwell, Aline Domingues, Mauricio A. Martins, John Pham, Daniela Weiskopf, John Altman, Esper G. Kallas, Kristian G. Andersen, Mario Stevenson, Paola Lichtenberger, Hyeryun Choe, Stephen S. Whitehead, Alessandro Sette, David I. Watkins

**Affiliations:** 1 Department of Pathology, University of Miami Miller School of Medicine, Miami, FL, United States of America; 2 Division of Vaccine Discovery, La Jolla Institute for Allergy and Immunology, La Jolla, CA, United States of America; 3 Department of Immunology and Microbial Science, The Scripps Research Institute, Jupiter, FL, United States of America; 4 Department of Immunology and Microbial Science, The Scripps Research Institute, La Jolla, CA, United States of America; 5 Division of Infectious Disease, University of Miami Miller School of Medicine, Miami, FL, United States of America; 6 Division of Clinical Immunology and Allergy, School of Medicine, University of São Paulo, São Paulo, SP, Brazil; 7 Department of Microbiology and Immunology and Emory Vaccine Research Center, Emory University, Atlanta, GA, United States of America; 8 National Institute of Allergy and Infectious Diseases, National Institutes of Health, Bethesda, MD, United States of America; Oregon Health and Science University, UNITED STATES

## Abstract

Zika virus (ZIKV) is a mosquito-borne flavivirus of significant public health concern. In the summer of 2016, ZIKV was first detected in the contiguous United States. Here we present one of the first cases of a locally acquired ZIKV infection in a dengue-naïve individual. We collected blood from a female with a maculopapular rash at day (D) 5 and D7 post onset of symptoms (POS) and we continued weekly blood draws out to D148 POS. To establish the ontogeny of the immune response against ZIKV, lymphocytes and plasma were analyzed in a longitudinal fashion. The plasmablast response peaked at D7 POS (19.6% of CD19^+^ B-cells) and was undetectable by D15 POS. ZIKV-specific IgM was present at D5 POS, peaked between D15 and D21 POS, and subsequently decreased. The ZIKV-specific IgG response, however, was not detected until D15 POS and continued to increase after that. Interestingly, even though the patient had never been infected with dengue virus (DENV), cross-reactive IgM and IgG binding against each of the four DENV serotypes could be detected. The highest plasma neutralization activity against ZIKV peaked between D15 and D21 POS, and even though DENV binding antibodies were present in the plasma of the patient, there was neither neutralization nor antibody dependent enhancement (ADE) of DENV. Interestingly, ADE against ZIKV arose at D48 POS and continued until the end of the study. CD4^+^ and CD8^+^ T-cells recognized ZIKV-NS2A and ZIKV-E, respectively. The tetramer positive CD8^+^ T-cell response peaked at D21 POS with elevated levels persisting for months. In summary, this is the first study to establish the timing of the ontogeny of the immune response against ZIKV.

## Author summary

Zika virus (ZIKV) is an emerging viral disease that has the potential to negatively impact future generations by causing birth defects in infected pregnant mothers. While there have been many studies performed in animal models of ZIKV infection, there have only been a limited number of reports studying the immune responses in humans. Ricciardi *et*. *al*. analyzed the immune response of a primary ZIKV infection in a dengue virus (DENV) naïve individual during the 2016 outbreak in Miami, Florida. B- and T-cell responses were assessed over multiple time points. Cross-reactive antibodies against DENV, a virus that the patient was never infected with, were generated during the ZIKV infection, but these antibodies failed to neutralize any of the DENV serotypes. Furthermore, while these DENV-cross-reactive antibodies might be expected to cause antibody dependent enhancement (ADE) of DENV infection, they did not. Interestingly, ADE of ZIKV infection was seen at approximately 1 ½ months after infection. Together, these results establish the timing of the ontogeny of the immune response against a primary ZIKV infection in a DENV-naïve individual.

## Introduction

The sudden emergence of Zika virus (ZIKV) cases in the Americas and the growing concern over the birth defects in ZIKV-infected pregnant mothers, led the World Health Organization (WHO) to declare ZIKV infection to be a Public Health Emergency of International Concern (PHEIC) on February 1^st^, 2016 [1–5]. ZIKV is a member of the *Flavivirdae* family along with other viruses including dengue virus (DENV), West Nile virus (WNV), and Yellow fever virus (YFV) [6, 7]. ZIKV, DENV, and YFV all share a common vector for transmission, the mosquito *Aedes aegypti* [8], and autochthonous human ZIKV infection was limited to Africa and mainland Asia until 2007 [4, 9]. Recent and continuous travel of infected humans has spread and established ZIKV infection to the Americas from Micronesia [3, 10, 11]. After infection with any of these flaviviruses, cross-reactive antibody responses are common [12, 13]. The cross-reactive antibody responses associated with primary and secondary DENV infections have been studied in depth [14–16]. The study of the ontogeny of cross-reactive antibodies after primary ZIKV infection is limited in flavivirus-naïve humans, however there have been several studies examining cross-reactive responses of ZIKV and DENV infections at single time points [17, 18].

ZIKV was first reported in the contiguous United States (US) on July 29^th^, 2016, when the Centers for Disease Control and Prevention (CDC) confirmed four locally acquired ZIKV infections in Miami, Florida (FL) [7, 19, 20]. Local, mosquito-borne ZIKV transmission, however, likely started in FL 2–3 months prior to detection [21]. Due to its warm and humid climate, Miami is conducive to year round breeding of the primary ZIKV vector, *Aedes aegypti* [8, 11, 22, 23]. This, along with the constant influx of tourists from ZIKV-endemic and ZIKV-naïve populations around the world, will most likely facilitate future ZIKV outbreaks in Miami [21].

Approximately 20% of ZIKV-infected individuals exhibit symptoms, making it difficult to study primary ZIKV infection without the complication of other co-circulating tropical diseases and flaviviruses [3, 24]. ZIKV-infected patients with symptoms often experience a mild febrile illness with fever and a rash, while other less common symptoms include pruritus, myalgia, and retro-orbital pain [3, 24, 25]. These patients will rarely visit a clinic for diagnosis or treatment, leaving the actual number of infected individuals unknown. In cases involving pregnant women, perinatal transmission to the fetus has been documented to result in microcephaly and other fetal complications [26–29]. Moreover, as the virus replicates in the brain tissue of the fetus, a wide array of cognitive developmental symptoms may potentially develop over time. Furthermore, ZIKV-infection has been associated with an increased risk of developing Guillain-Barré syndrome; however, the exact mechanism is not yet fully understood [30–32]. While there are many factors that contribute to disease outcomes, understanding the ontogeny of the immune response to primary ZIKV infection could help the evaluation of diagnostics, vaccines, and other therapies.

Early during the outbreak, we discovered an individual with symptoms consistent with ZIKV infection in Miami. She had no international travel history and was thus suspected of locally acquiring ZIKV. We confirmed ZIKV infection in this individual using RT-PCR from blood and saliva, and we followed the development of her immune response from day (D) 5 post onset of symptoms (POS) to D148 POS. Here, for the first time, we describe a detailed ontogeny of the plasmablast, antibody, and T-cell immune responses in one of the first locally acquired ZIKV infections in the contiguous US.

## Methods

### Ethics statement

Research on human subjects was conducted in compliance with DoD, NIH, federal, and state statutes and regulations relating to the protection of human subjects and adheres to principles identified in the Belmont Report (1979). All human subjects were consented in writing and all specimens, data, and human subject research were gathered and conducted for this publication under University of Miami IRB-approved protocol study number 20160127. Written consent was provided for the use of photos from the patient.

### Patient

Blood samples were collected from volunteer Hu0015, a 34-year-old woman who reported a skin rash that started five days prior to the first blood draw. Plasma and PBMCs were obtained from blood samples collected in ethylenediaminetetraacetic acid (EDTA) tubes, as well as saliva in sterile 50 mL conical tubes, at day (D) 5, 7, 15, 21, 28, 48, 56, 70, 91, 106, 116, and 148 post onset of symptoms (POS). ZIKV infection was confirmed using reverse-transcriptase (RT) PCR assay for ZIKV RNA in the D5 POS saliva sample as well as plasma samples collected at D5 and D7 post onset of the first rash symptoms. No previous history of DENV infection was reported by the volunteer. YFV vaccination status was unknown to the patient, but because she had never traveled outside of the United States, it is unlikely that she was ever vaccinated against YFV. Whole blood was sent to HistoGenetics LLC (http://www.histogenetics.com) for MHC typing.

### ZIKV quantification and sequencing

Viral RNA was extracted using the RNAeasy kit (QIAGEN). ZIKV genome equivalents (GE) were calculated using a qRT-PCR assay targeting the nonstructural protein 5 region (9014–9123 nt), as described [21]. ZIKV sequencing was performed using an amplicon-based approach [21, 33]. Briefly, cDNA was reverse transcribed from 5 μl of RNA using SuperScript IV (Invitrogen). ZIKV cDNA (2.5 μl/reaction) was amplified in 35 × 400 bp fragments from two multiplexed PCR reactions using Q5 DNA High-fidelity Polymerase (New England Biolabs). The amplified ZIKV cDNA fragments (50 ng) were prepared for sequencing using the Kapa Hyper prep kit (Kapa Biosystems) and SureSelect XT2 indexes (Agilent). Agencourt AMPure XP beads (Beckman Coulter) were used for all purification steps. Paired-end 251 nt reads were generated on the MiSeq using the V2 500 cycle kit (Illumina). Demultiplexing was performed by the Illumina instrument. The primer sequences were removed from the reads and bases with Phred quality scores < 20 were removed by Trimmomatic [34]. The reads were then aligned to the complete genome of a ZIKV isolate from the Dominican Republic, 2016 (GenBank: KU853012) using Novoalign v3.04.04 (www.novocraft.com). Samtools was used to sort the aligned BAM files [35]. ZIKV-aligned reads were visually inspected using Geneious v9.1.5 before generating consensus sequences [36]. The consensus sequence for sample Hu0015Sa is available on GenBank (KX832731).

### Phylogenetic analysis

Published ZIKV genomes (195) from the Pacific and the Americas (Asian genotype, from 2013–2016) were retrieved from GenBank and from recent large sequencing projects [21, 37]. The protein-coding sequences were aligned together with the Hu0015 genome using MAFFT [38]. A maximum likelihood phylogenetic tree was reconstructed with RAxML using the general time-reversible (GTR) nucleotide substitution model and gamma distributed rates amongst sites [39, 40]. The phylogenetic tree was annotated using ETE Toolkit [41].

### Plasmablast staining

We determined the frequency of plasmablasts in circulation by flow cytometric analysis of PBMCs obtained from blood collected in EDTA tubes and used a Ficoll-Paque (GE Lifesciences) gradient for separation. Briefly, we stained fresh PBMC samples (1 x 10^6^ cells, room temperature, in the dark), with 100 μl of a surface antibody cocktail (S1 Table). We also included the fixable viability dye LIVE/DEAD Fixable Red Dead Cell Stain Kit (Life Technologies) in the staining mix, in order to discriminate between live and dead cells. After 30 min, we washed the cells twice with FACS buffer (PBS, 0.5% FBS, 2 mM EDTA) and resuspended with a PBS 1x solution. Samples were acquired the same day using either a SONY SH800 or a BD FACSAria IIu flow cytometer and analyzed using FlowJo 9 (Tree Star Software).

### Virus stocks

The DENV1 (strain West Pac74; GenBank U88535.1), DENV2 (strain New Guinea C; GenBank AF038403.1), DENV3 (strain Sleman/78; GenBank AY648961), DENV4 (strain Dominica/8129; GenBank AF326573.1), and ZIKV (strain Paraiba/2015; GenBank KX280026) were propagated in Vero cells (ATCC). Virus stocks were used for both binding virus capture assays (VCA) and neutralization assays as described below. ZIKV (strain PB-81) was gifted from The World Reference Center for Emerging Viruses and Arboviruses (WRCEVA) at The University of Texas Medical Branch (UTMB). ZIKV (strain PB-81) was used for all antibody dependent enhancement experiments and was also propagated in Vero cells.

### Virus Capture Assay (VCA)

Antibody and plasma binding was determined in a side-by-side DENV1, DENV2, DENV3, DENV4, and ZIKV VCA ELISA. The ELISA plate was coated with the mouse-anti-flavivirus monoclonal antibody 4G2 (clone D1-4G2-4-15, EMD Millipore) diluted 1:1,000 in carbonate binding buffer and incubated overnight at 4°C. The next day, the plate was washed 5-times with PBS-Tween20 and the wells were blocked with 5% skim milk in PBS for 1 h at 37°C. Following the block, the plate was washed and each virus was added to the corresponding VCA wells, respectively, and incubated for 1 h at room temperature. Subsequently, the plate was washed with PBS and plasma from different time points diluted in 5% skim milk were added to designated wells and incubated for 1 h at 37°C. Following sample addition, plates were washed and detection was carried out using the antibody goat anti-human IgG HRP (SouthernBiotech, 2045–05) diluted 1:10,000, was added to all wells and incubated for 1 h at 37°C. The plate was washed and the wells were developed with the TMB substrate at room temperature for 3–4 min. The reaction was then stopped with the TMB solution, and absorbance was read at 450 nm.

### Flow cytometry-based neutralization assay

The neutralizing potency of human plasma was measured using a flow cytometry-based neutralization assay (NEUT) [42, 43]. In brief, human EDTA-plasma was diluted and pre-incubated with the reference ZIKV or DENV serotypes in a final volume of 220 μl for 1 h at 37°C. The virus and plasma mixture (100 μl) was added onto wells of a 24-well plate of 100% confluent Vero cell monolayers in duplicate. The inoculum was incubated in a 37°C incubator at 5% CO_2_ for 1 h with agitation of the plates every 15 min. After 1 h, the virus and plasma inoculums were aspirated and the wells were washed with media. Fresh media was then added and the plates were incubated for a total of 24 h. Cells were trypsinized with 0.5% trypsin (Life Technologies), fixed (Cytofix; BD), and permeabilized (Cytoperm; BD). Viral infection was detected with the 4G2 antibody (clone D1-4G2-4-15, EMD Millipore) recognizing ZIKV or DENV, followed by staining with an anti-mouse IgG2a APC fluorophore-conjugated secondary reagent (clone RMG2a-62; Biolegend). The concentration to achieve half-maximal neutralization (NEUT_50_) was calculated using a nonlinear regression analysis with Prism 7.0 software (GraphPad Software, Inc.).

### Plaque reduction neutralization test

Plaque reduction neutralization tests (PRNTs) were conducted as previously described [44]. Briefly, human plasma was serially diluted in OptiMEM supplemented with 2% human serum albumin, 2% fetal bovine serum, and gentamicin. Virus was diluted to a final concentration of approximately 500–1,000 PFU/mL in the same diluent and was added to equal volumes of the diluted plasma and mixed. The virus/plasma mixture was incubated at 37°C for 30 min. Cell culture medium was removed from 90% confluent monolayer cultures of Vero cells on 24-well plates and 100 μl of the virus/plasma mixture was transferred onto duplicate cell monolayers. Cell monolayers were incubated for 60 min at 37°C and overlaid with 1% methylcellulose in OptiMEM supplemented with 2% FBS, 2 mM glutamine, and 50 μg/mL gentamicin. Samples were incubated at 37°C for four days after which plaques were visualized by immunoperoxidase staining, and a 50% plaque-reduction neutralization titer (PRNT_50_) was calculated.

### Antibody dependent enhancement

Human plasma was serially diluted in RPMI media containing 10% FBS and was mixed with either 1.5 x 10^3^ PFU of DENV2 or ZIKV. The plasma and virus mixture, in a total of 50 μl, was incubated for 1 h at 37°C. The mixture was then added to 5 x 10^3^ K562 cells in 50 μl of RPMI containing 10% FBS in a 96-well plate. Three days later, the cells were fixed, permeabilized, and stained with a pan-flavivirus antibody 4G2 as previously described [45]. Samples were analyzed with an Accuri C6 flow cytometer.

### Peptide selection and synthesis

15-mer peptides overlapping by 10 amino acids (aa) spanning the whole ZIKV polyprotein were synthesized (A & A/Synthetic Biomolecules). Subsequently, peptides were combined in 10 Mega-pools according to the ZIKV protein from which they were derived as previously described [46]. Pools that elicited an IFN-γ response were subsequently deconvoluted to identify the individual peptide inducing the response.

The ZIKV 15-mer peptide EPRTGLDFSDLYYLT was identified to induce a ZIKV-specific CD8^+^ T-cell response. To establish a minimal optimal epitope, all of the possible 8-, 9-, 10-, and 12-mer peptides that had potential to bind any of the MHC class I molecules present in the donor were synthesized and tested. Epitope predictions for class I were made using the consensus prediction methods publicly available through the IEDB Analysis Resource (www.iedb.org).

### Intracellular cytokine staining

PBMCs (1 × 10^6^ cells/well) were incubated with ZIKV mega-pools (1 μg/mL) for 6 h. After 2 h of stimulation, Brefeldin A (1 μg/mL; BD Bioscience) was added. At the end of the stimulation, cells were washed, and stained for 30 min with primary antibody cocktail. After primary surface staining, cells were washed, fixed with 4% paraformaldehyde, permeabilized, blocked with normal human sera (Gemini), and stained for intracellular IFN-γ and Granzyme B. A BD LSR-II flow cytometer was used for data acquisition (BD) and data were analyzed with FlowJo X software (Tree Star Software). A full list of antibodies used in the ICS staining are shown in supplementary (S1 Table).

### HLA A*01:01 tetramer staining

The GLDFSDLYY 9-mer sequence was used to synthesize an A*01:01 tetramer by the National Institute of Health (NIH) Tetramer Core Facility. For tetramer staining, PBMCs (2 × 10^6^ cells/well) were incubated with the tetramer (1:100 dilution) for 90 min at room temperature. After 1 h of incubation with tetramer, surface antibodies were added (S1 Table) for half an hour at room temperature. Cells were then washed, fixed, acquired, and analyzed as previously described [47, 48].

### IFN-γ ELISPOT assay

2 × 10^5^ PBMCs were incubated in triplicates with 0.1 mL complete RPMI 1640 in the presence of individual peptides at different final concentrations [1, 0.1, 0.01, and 0.001 μg/ml]. Following a 20 h incubation at 37°C, the cells were discarded, the wells were incubated with biotinylated IFN-γ mAb (clone 7-B6-1; Mabtech) for 2 h, and the wells were developed as previously described [47, 48].

## Results

### Patient

**Fig 1:**
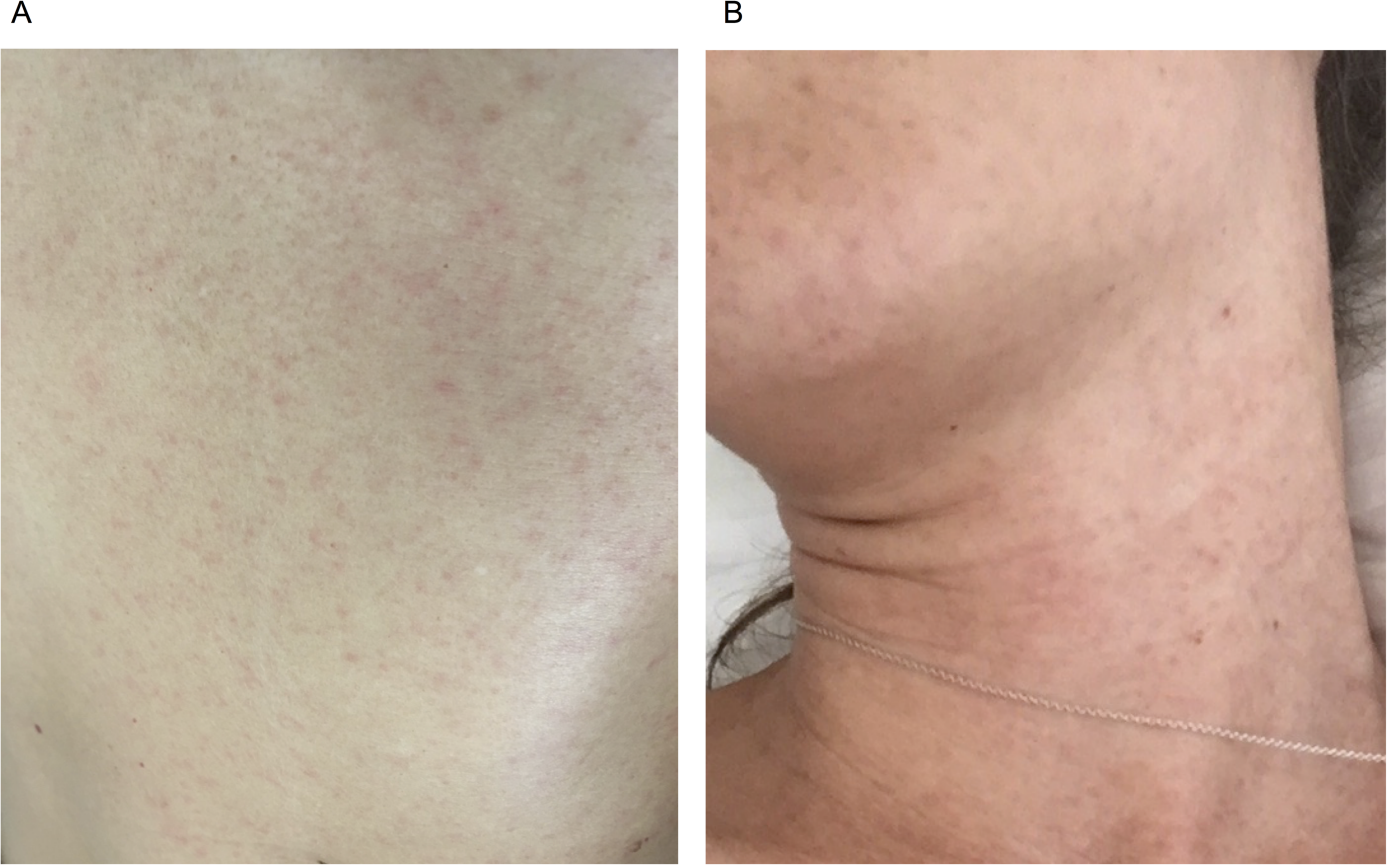
ZIKV induced maculopapular rash. Upon admission into the study photos of the patient’s rash were taken D3 POS. This rash was neither itchy, nor sensitive to the touch. (A) A close-up of the initiation point of the rash on the front of the torso near the midline. (B) The rash then spread from the torso to the neck and head.

During the ZIKV outbreak in Miami, FL in 2016, a female subject (Hu0015) noticed an abnormal rash, which started on the torso and continued to spread centrifugally, eventually reaching her extremities. The rash (Fig 1) was neither itchy nor sensitive. Mild general malaise was also reported. She did not report pruritus, myalgia, fever, retro-orbital pain, or GI symptoms and was not hospitalized. Immediate speculation of ZIKV infection prompted the study team to enroll Hu0015 in a University of Miami protocol approved by the Human Subjects Board to characterize ZIKV infection. In total, we collected blood and saliva at D5, 7, 15, 21, 28, 48, 56, 70, 91, 106, 116, and 148 POS. We separated whole blood into peripheral blood mononuclear cells (PBMCs) and EDTA-plasma for all time points. While most of the samples were immediately frozen for later analysis, we stained fresh PBMCs from D5, 7, 15, 21, and 148 POS for the human plasmablast (Pb) phenotype in order to track Pb development.

**Fig 2:**
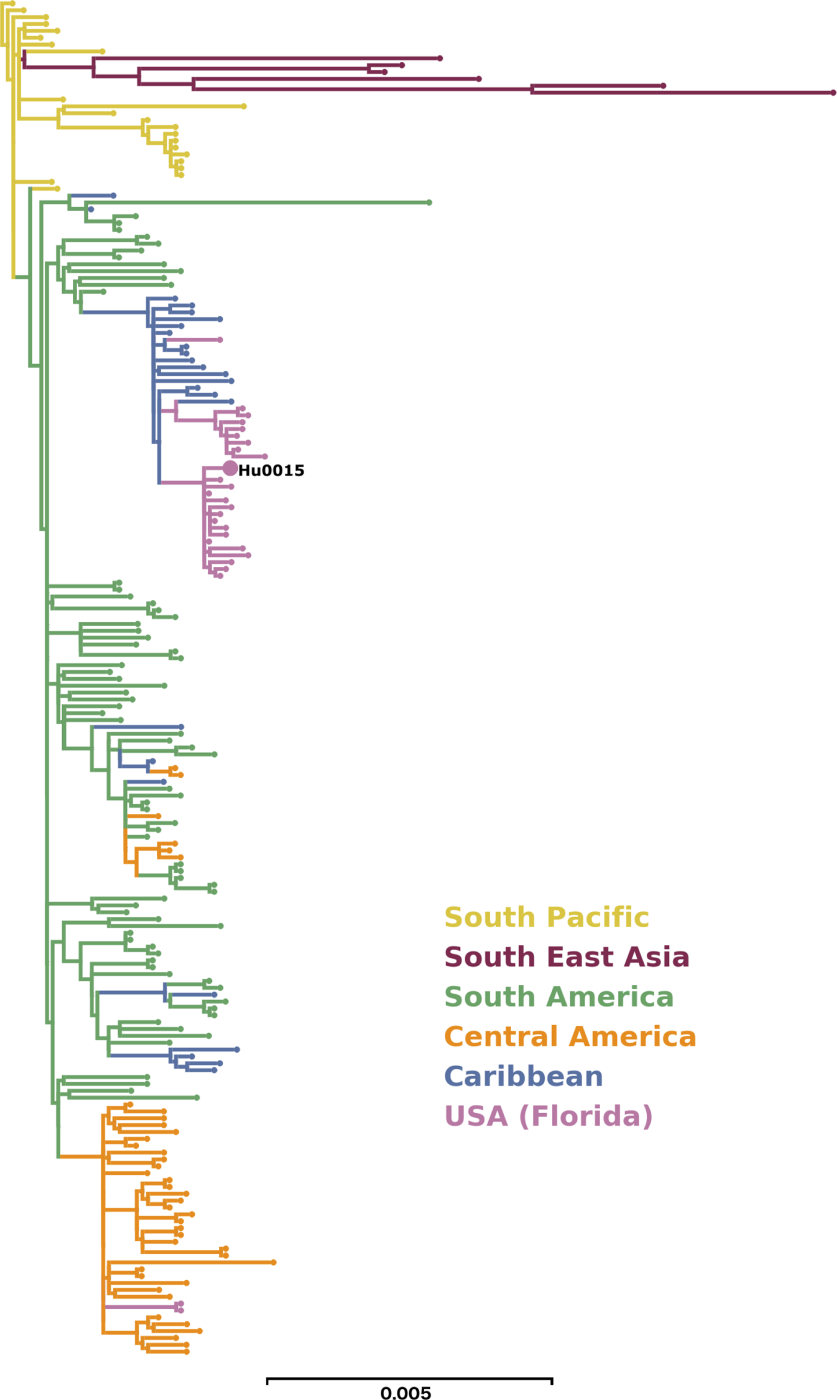
Phylogenetic tree of ZIKV isolated from Hu0015 compared to previously sequenced ZIKV genomes. A detailed maximum likelihood phylogenetic analysis of published ZIKV genomes from the Pacific and Americas (Asian genotype, from 2013–2016). The pink branches represent isolates from the 2016 ZIKV outbreak in Florida. Hu0015 is one of the first ZIKV sequences from autochthonous transmission in the contiguous US and clades with one of four ZIKV lineages detected during the outbreak in Florida. The scale of 0.005 represents nucleotide substitutions per site in the viral genome.

We extracted RNA from plasma at D5, 7, and 15 POS to isolate and quantify virus. We saw RT-PCR amplification with ZIKV-specific probes in the D5 and D7 POS plasma samples in the range of 150 genome equivalents (GE)/mL, but amplification from the D15 POS plasma sample was not detected. The D5 POS saliva sample yielded 21,610 GE/mL, but ZIKV was undetectable in saliva collected at D7 and D15 POS. We sequenced the D5 saliva and produced > 4.5 million 250 nucleotide (nt) sequences aligning to the ZIKV genome [33]. These sequences formed a 10,609 nt contig that covered 100% of the protein-coding sequence and 98.2% of the ZIKV genome at an average depth of 93,103 nt. The results have been deposited into GenBank (Accession ID KX832731). We reconstructed a maximum likelihood phylogenetic tree using the ZIKV genome from Hu0015 and from 195 published genomes from the Pacific and the Americas since 2013 (Fig 2). The placement of the ZIKV genome from subject Hu0015 with other ZIKV genomes recovered from the Miami outbreak confirms that this was a locally-acquired infection.

Subject Hu0015 expressed the MHC-I alleles A*01:*01*:*01*, A*02:*01*:*01*, B*08:*01*:*01*, and B*41:*02*:*01* (Table 1). The MHC-C alleles were not typed. Hu0015’s MHC-II alleles were DRB1*13:*03*:*01*, DRB1*15:*02*:*01*, DQB1*03:*01*:*01*, DQB1*06:*01*:*01*, DPB1*11:*01*:*01*, and DPB1*13:*01*:*01* (Table 1).

**Table 1:** Patient information.

Identification				Diagnosis		Medical history	MHC Class I Typing	MHC Class II Typing	Initial Symptoms^a^ (D0)
ID	City	Sex	Age	Plasma	Saliva				
Hu0015	Miami	F	32	RT-PCR positive D5^a^ & D7^a^	RT-PCR positive D5^a^	No dengue fever, unknown YF vaccination	A*01:01:01 A*02:01:01 B*08:01:01 B*41:02:01	DRB1*13:03:01 DRB1*15:02:01 DQB1*03:01:01 DQB1*06:01:01 DPB1*11:01:01 DPB1*13:01:01	Maculopapular rash of the torso, general malaise

^a^ Time point after onset of symptoms.

### Plasmablast response

**Fig 3:**
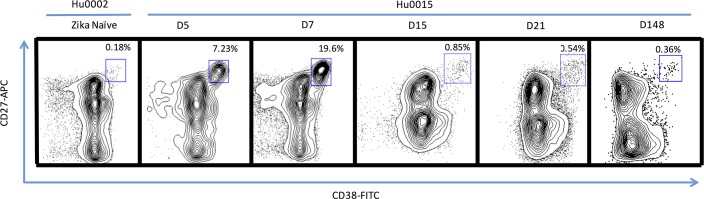
Ontogeny of the plasmablast response of a primary ZIKV infection in a flavivirus-naïve individual. The blue box in each flow graph represents the frequency of Pbs from isolated PBMCs as a percentage of total CD19^+^ B-Cells. The Pb frequency was significantly elevated at D5 POS when compared to a naïve uninfected individual as well as to convalescent phase PBMCs from Hu0015 (D148 POS).

We first determined the timing of the emergence of the Pb response after ZIKV infection. In the case of other flavivirus infections, the induction of massive Pb responses D5 to D14 POS has been reported [49, 50]. We stained Hu0015 PBMC for the human Pb phenotype (CD3^−^/CD19^+^/CD20^−/low^/CD38^high^/CD27^high^) and expressed this as a fraction of the total CD19^+^ B-cells. Pb frequency was measured at D5, 7, 15, 21, and 148 POS along with samples from a ZIKV- and DENV-naïve individual as a control. At D5 POS, 7.23% of the CD19^+^ B-cells expressed the Pb phenotype while only 0.18% Pbs were present in the naïve control (Fig 3). As the immune response progressed, the Pb frequency increased to 19.6% at D7 POS (Fig 3). The Pb frequency contracted to a baseline level of 0.85% and 0.54% at D15 and D21 POS respectively (Fig 3). The frequency of circulating Pbs during the convalescent phase, measured at D148 POS, was 0.36%. This measurement served as a baseline control for Hu0015.

### Antibody response

**Fig 4:**
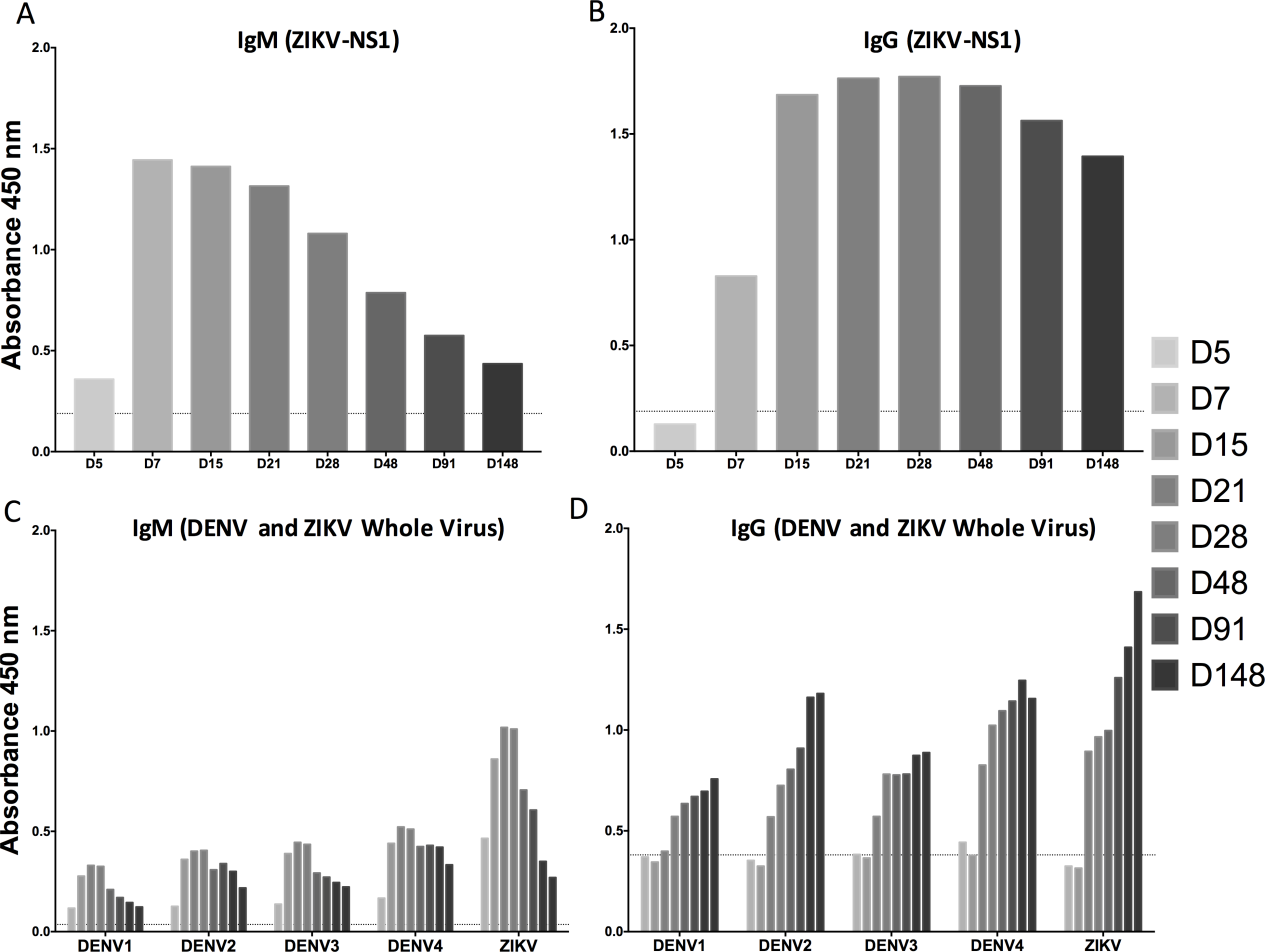
ZIKV-specific and DENV-cross-reactive antibody responses over time. (A) IgM binding against recombinant ZIKV-NS1 from Hu0015 plasma showed minimal binding at D5 POS, but increased rapidly and peaked at D7 POS. (B) The ZIKV-specific NS1 IgG response shows little to no ZIKV-specific NS1 IgG antibody present at D5 POS, but a large increase at D15 POS, with levels remaining elevated out to the last time point measured at D148 POS. (C) DENV-cross-reactive IgM was present soon after infection. However, these levels were not as high as the IgM response against ZIKV. These titers rose and fell, with the peak between D15 and D21 POS. (D) DENV-cross-reactive IgG was present soon after infection, however the levels were not as high as the IgG response against ZIKV. The titers against DENV and ZIKV experienced an increase between D7 and D15 POS. IgG levels continued to rise throughout infection and were the highest at the last time point measured at D148 POS. The dotted line is background binding from an uninfected, flavivirus-naïve individual.

The non-structural 1 (NS1) protein is secreted into the circulation early on during flavivirus infection [6]. We, therefore, assessed plasma from subject Hu0015 for IgM binding against recombinant ZIKV-NS1 protein (Fig 4A). At D5 POS we observed low levels of ZIKV-NS1-specific IgM antibodies. However, at D7 POS, there was a dramatic rise. The peak of the ZIKV-NS1-specific IgM response occurred between D7 and 15 POS, and then the ZIKV-NS1-specific IgM response declined over time, reaching baseline levels by D148 POS. We then assessed the ZIKV-NS1-specific IgG response (Fig 4B). There was no ZIKV-NS1-specific IgG reactivity at D5 POS. At D7 POS, ZIKV-specific NS1 IgG level had increased by over 300% from D5 POS and continued to increase until D15 POS. From D15 to D48 POS, ZIKV-specific NS1 IgG antibodies were relatively stable in the plasma. At D91 POS there was a decrease, with another slight decrease at D148 POS.

Considerable cross-reactivity among flavivirus-specific antibodies and the various members of the flavivirus family has been reported [13, 17, 51–55]. We sought to understand the exact timing of the appearance of this cross-reactivity after ZIKV infection of a previously DENV-naïve patient. Hu0015 had no recent travel history outside the US and there was no evidence that she had any detectable pre-existing anti-DENV IgG binding antibodies at either D5 or D7 POS. We analyzed DENV-cross-reactive IgM and IgG antibodies from Hu0015 using whole virus binding enzyme-linked immunosorbent assays (ELISAs). We assessed DENV1-4 binding and demonstrated that the initial IgM response was already cross-reactive by D5 (Fig 4C). Even though cross-reactive DENV IgM responses were present at D5 POS, the levels of these IgG antibodies were less than those that were directed against ZIKV. The peak of the IgM response directed against the four DENV serotypes occurred between D15 and D21 POS and it diminished after that. The peak of the DENV IgM response was lower than that against ZIKV. As expected, the initial IgG response against both DENV and ZIKV was delayed in comparison to the IgM response. The DENV-cross-reactive IgG response against serotypes 1–4 was low at the early time points (Fig 4D). However, with time, cross-reactive DENV1-4 IgG responses continued to rise, albeit not as rapidly as the response against ZIKV. Interestingly, antibodies in the plasma from Hu0015 appeared to bind DENV1 and DENV3 less than DENV2 and DENV4.

**Fig 5:**
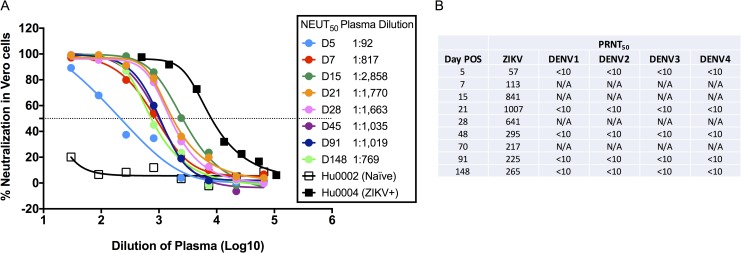
Neutralization titers against ZIKV and DENV. (A) Neutralization titers were performed by flow cytometry at several time points against ZIKV-Paraiba/2015 and the NEUT_50_ was calculated based on a non-linear regression. Peak NEUT_50_ occurred at D15 POS. Hu0002 was used as a flavivirus-naïve control and Hu0004 was a DENV- and ZIKV-exposed control. (B) Plaque reduction neutralization tests (PRNTs) were also performed against ZIKV and all four DENV serotypes. PRNT_50_ was calculated as 50% neutralization of plaques based on control virus wells and reported as a dilution of patient plasma. (N/A = samples not run).

We then determined when ZIKV-specific neutralization capacity developed in our patient. We conducted neutralization experiments using flow cytometry (NEUT) to assess the ability of plasma to neutralize the ZIKV-Paraiba strain (Fig 5A). We used Hu0002, a ZIKV- and DENV-seronegative subject, and Hu0004, a ZIKV- and DENV-seropositive individual as controls. Hu0015, at D5 POS, neutralized ZIKV, but this only occurred at low dilutions. At D7 POS, the NEUT 50% neutralization point (NEUT_50_) increased almost 10-fold to 1:817. The plasma NEUT_50_ titer peaked at D15 POS at 1:2,858 and the titer remained high through D48 POS. Plaque reduction neutralization tests (PRNTs) were also performed against the ZIKV-Paraiba strain out to D148 POS. The calculated 50% neutralization point for plaques (PRNT_50_), yielded similar titers to the NEUT_50_ titers. NEUT_50_ titers were slightly higher than the PRNT_50_ titers. PRNTs were also performed for all DENV serotypes for the selected time points (Fig 5B). Surprisingly, Hu0015 plasma did not neutralize any of the DENV serotypes at any time point, despite the presence of binding antibodies (Fig 5B).

**Fig 6:**
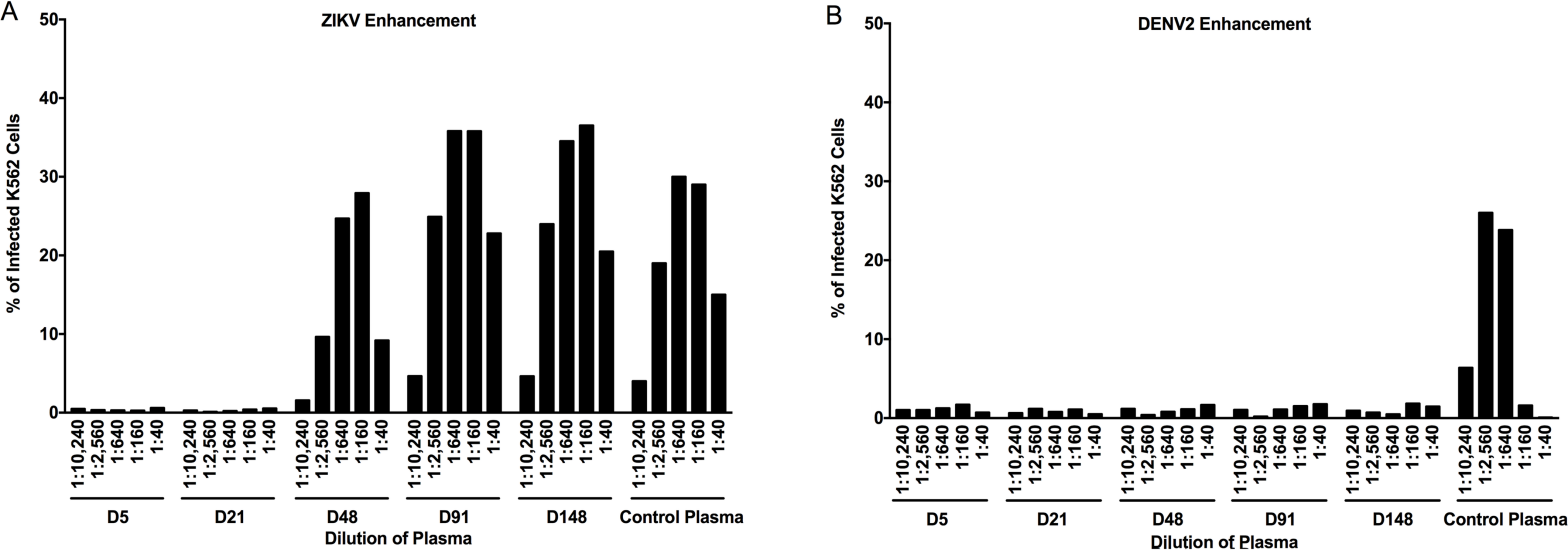
Antibody dependent enhancement of ZIKV but not DENV2. (A) ADE was performed by flow cytometry at several time points against ZIKV BR-81 at an MOI of 0.3 with select time points from Hu0015’s plasma. Enhancement did not occur until D48 POS and was seen until the last time point measured (D148 POS). (B) ADE against DENV2 at an MOI of 0.3 was also performed. Enhancement was not seen at any time points. Control monoclonal virus-specific antibodies were used in both panels and yielded a minimum of 20% infection in each assay. Human plasma that enhanced ZIKV or DENV2 in K562 cells was used as a positive control in each assay.

Since there was no neutralization of DENV with the patient’s plasma, we sought to determine whether the patient’s plasma could mediate antibody dependent enhancement (ADE) using K562 cells. These immortalized monocyte lineage cells are not permissive to DENV or ZIKV infection, but express the Fc-gamma receptor (FcR), thereby facilitating flavivirus infection. Patient plasma from D5 and D21 POS showed no enhancement of ZIKV (Fig 6A). However, at D48 POS, the patient’s plasma enhanced ZIKV infection and this continued until the last time point measured (D148) (Fig 6A). Peak ADE occurred at dilutions of plasma between 1:160 and 1:640. Surprisingly, we saw no enhancement of DENV2 infection with the ZIKV-infected patient’s plasma at any time point measured (Fig 6B).

### T-cell response

**Fig 7:**
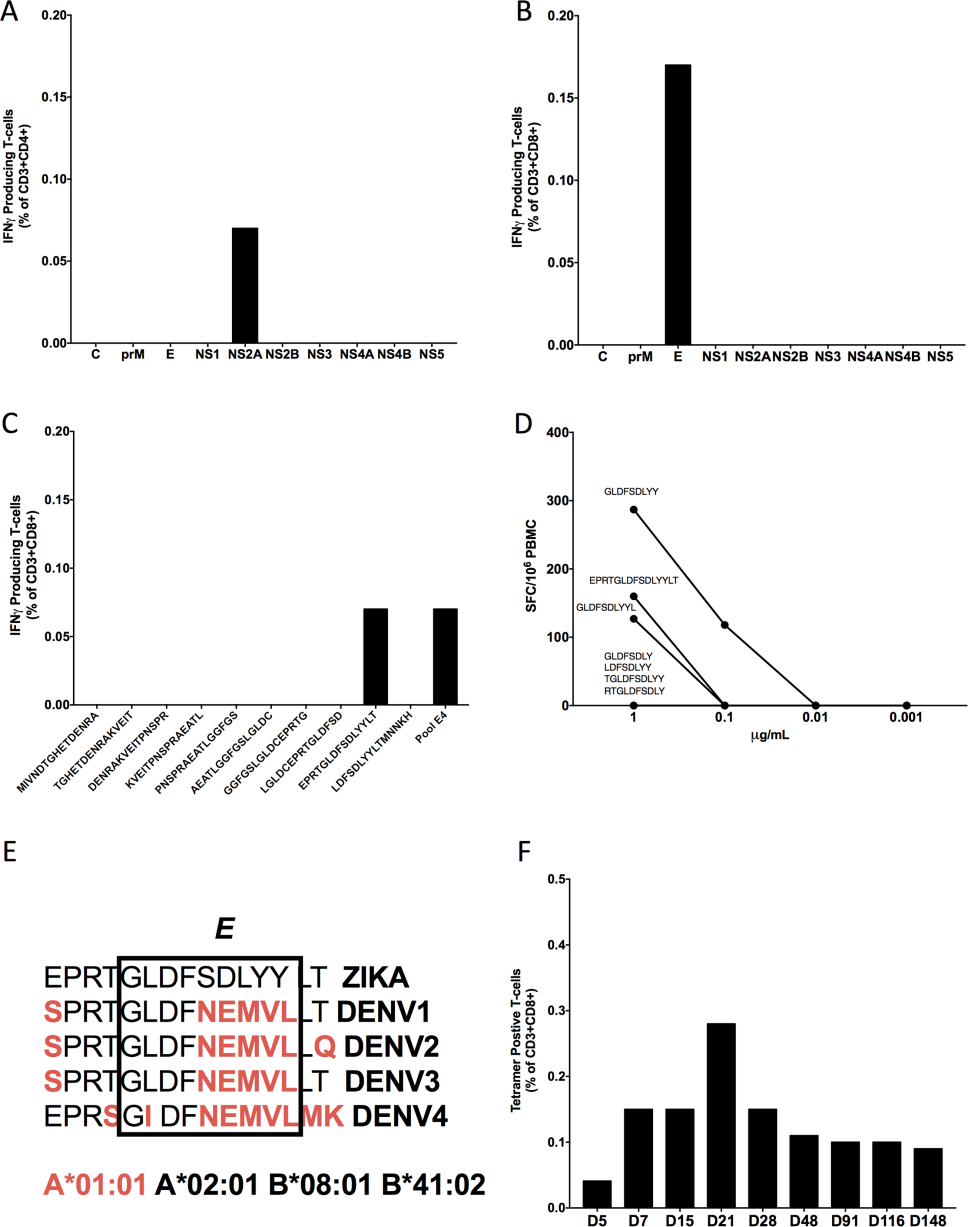
Ontogeny of the T-cell response. (A) Using PBMCs from D106 POS and mega-pools consisting of overlapping 15-mer peptides from each protein of ZIKV in ICS assays, we observed that the CD4^+^ T-cells responded to ZIKV-NS2A. (B) The CD8^+^ T-cells responded to ZIKV-E. (C) To further analyze the CD8^+^ T-cell response, the peptide pool that was responsible for the ZIKV-E response was fine mapped in ICS assays into ten individual 15-mer peptides. The response was directed against only one of the 15-mer peptides. (D) Using this peptide, epitope predictions for the patient’s MHC Class I alleles were made for all possible 8-, 9-, 10-, and 12-mer peptides. These peptides were synthesized and tested in and IFN-γ ELISPOT dilution assay. (E) We compared an alignment of the 9-mer minimal optimal peptide to ZIKV-E and to DENV-E. The amino acids, indicated in red, are amino acid differences from the reference ZIKV sequence. (F) The minimal optimal peptide was then used to make a tetramer, and this was used to track the ontogeny of ZIKV-specific CD8^+^ T-cell in this patient. A tetramer response was present at D5 POS, with a peak at D21 POS, and then remained level from D48 to D148 POS.

We were then interested in the ontogeny of the T-cell response against ZIKV. We used pools of overlapping 15-mer peptides spanning the entire ZIKV proteome in both intracellular cytokine staining (ICS) and IFN-γ ELISPOT analysis. Using ICS, ZIKV-specific CD4^+^ T-cell responses were detected against the ZIKV-NS2A protein (Fig 7A), and CD8^+^ T-cell responses were detected against the ZIKV-Envelope (E) protein (Fig 7B). Deconvolution of all ZIKV-E derived peptides identified the 15-mer EPRTGLDFSDLYYLT as a target of the ZIKV-specific CD8^+^ T-cell responses (Fig 7C). To establish the minimal optimal epitope, all possible 8-, 9-, 10-, and 12-mer peptides that had potential to bind any of the MHC class I molecules present in the donor were synthesized and tested with ELISPOT. The 9-mer GLDFSDLYY peptide was shown to elicit the strongest response of all peptides tested and the sequence of this peptide was consistent with the peptide binding motif for HLA-A*01:01 (Fig 7D). This 9-mer minimal-optimal peptide was then synthesized with a HLA-A*01:01 tetramer at the NIH Tetramer Core Facility. Interestingly, the first four amino acids of this 9-mer minimal-optimal peptide were conserved in all four DENV serotypes and ZIKV. The second half was unique to ZIKV, and was identical for all four DENV serotypes (Fig 7E). We then used this tetramer to track the ZIKV-specific response in the CD8^+^ T-cell compartment (Fig 7F). The initial tetramer response appeared at D7 POS, peaked at D21 POS, and was still present, albeit at a very low level, at D148 POS. The tetramer positive CD8^+^ T-cells over time can also be seen overlaid on top of the naïve, effector memory, central memory, and effector memory CD8^+^ T-cells based on expression of CCR7 and CD45RA markers (S1 Fig). Phenotypic analysis of the responding cells revealed that most tetramer positive cells were contained within the effector memory (CCR7^-^/CD45RA^-^) T-cell subset. An in-depth characterization of the phenotype of CD8^+^ T-cells was also performed by ICS (S2 Fig). We also analyzed the ontogeny of the CD8^+^ T-cell response by IFN-γ ICS against ZIKV protein-specific mega-pools. We saw very low levels (borderline to undetectable) of IFN-γ production at all time points except for D21 POS (S3 Fig), which matched the peak of our tetramer data. The ICS of the CD8^+^ IFN-γ^+^ T-cells at D21 POS revealed an overexpression of cytotoxic markers PD1 and Granzyme B, and a downregulation of CX3CR1. The controls for the flow cytometry experiments in Fig 7 were also performed with a ZIKV-naïve individual (S4 Fig).

## Discussion

**Fig 8:**
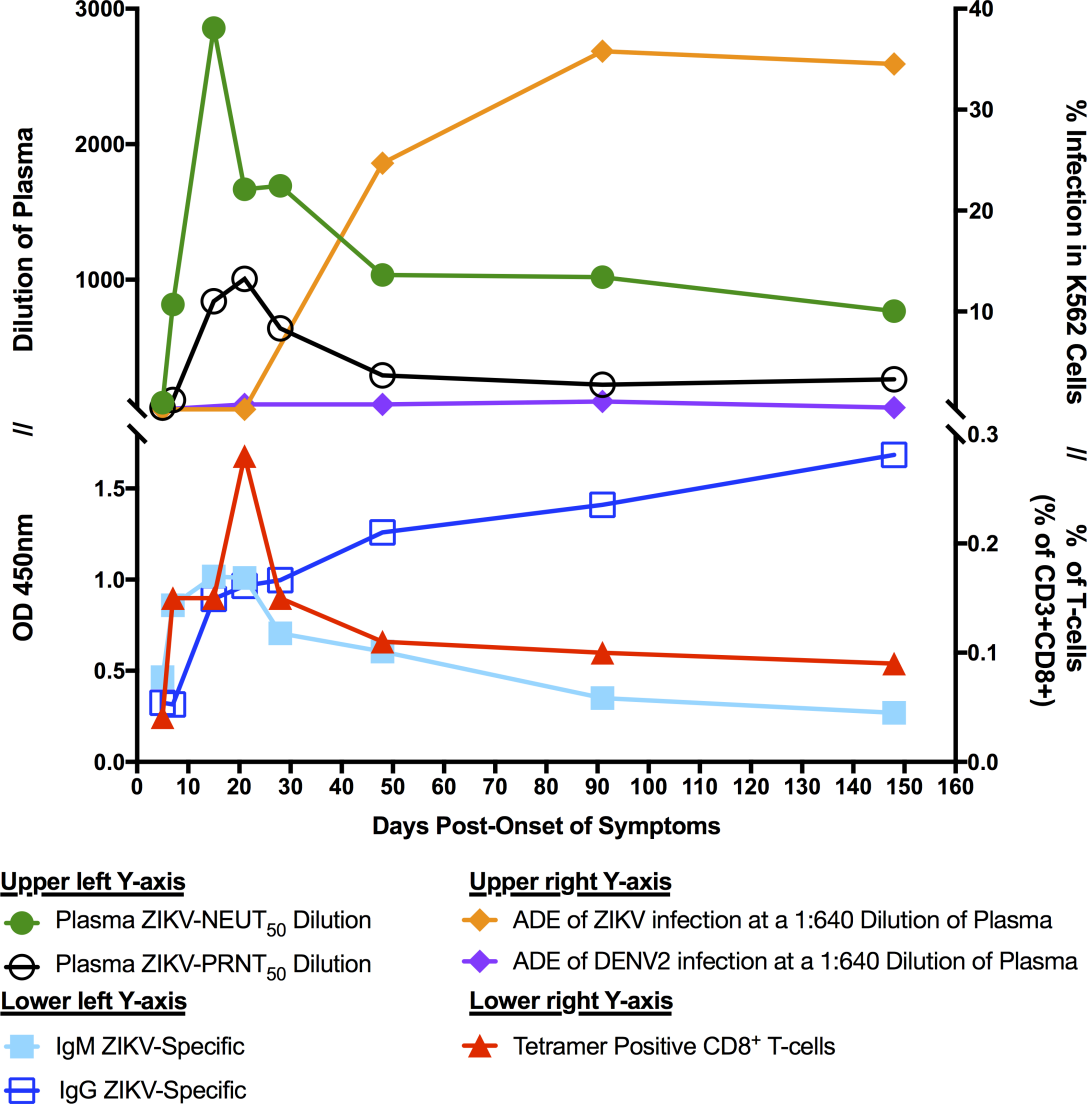
Summary of immune responses in a flavivirus-naïve, primary ZIKV infection. Antibody and T-cell responses were charted together to provide a view of the responses over time.

Local transmission of ZIKV has only recently been reported in the continental US [19]. From January, 2015 to May, 2017, the CDC has confirmed the diagnosis of over 5,000 ZIKV cases in the contiguous US, with 256 of these cases being local transmissions in Miami, FL [56]. Early during the outbreak, we discovered a patient exhibiting the hallmarks of ZIKV infection. This individual was confirmed by RT-PCR to have been infected with ZIKV and we followed her immune response until D148 POS. For the first time in humans, we describe a longitudinal study of the ontogeny of the plasmablast, antibody, and T-cell immune responses in one of the first locally-acquired ZIKV infections in the contiguous US (Fig 8).

The ZIKV isolated from this patient’s saliva was one of the first sequenced ZIKV genomes from an autochthonous mosquito-borne transmission in the US. Sexual transmission was ruled out, as the sole sexual partner of Hu0015 was also tested for ZIKV, and he was found to be negative for ZIKV by RT-PCR and anti-ZIKV antibodies by VCA. The phylogenetic placement of this ZIKV genome puts it close to other genomes sampled from the Wynwood and Miami Beach transmission zones later identified by the Florida Department of Health during the 2016 outbreak in Florida. Interestingly, the ZIKV isolate from Hu0015 was distinctly different from those circulating elsewhere in the Americas [21], which strongly suggests that the patient was indeed infected in Florida. Phylogenetic analysis indicates that the current Zika virus outbreak in Florida was the result of an imported infection (human or mosquito) from the ongoing epidemic in the Caribbean [21]. The Hu0015 ZIKV genome also clusters with genomes obtained from infected *Aedes aegypti* mosquitoes collected in Miami Beach, suggesting that the route of infection was likely mosquito-borne [21].

ZIKV infection, in mice and non-human primates (NHPs), has been well characterized, allowing investigators to follow the ontogeny of the immune response as well as analyze tissues not previously analyzed in humans [18, 57–61]. Mouse models can be useful in understanding cell signaling pathways in ZIKV infection as well as for rapidly screening drugs that control or prevent ZIKV replication [62, 63]. In addition, because ZIKV infection can induce fetal abnormalities in mice, they provide a more economical option than NHPs when initially developing prophylactic and therapeutic approaches to counteract the impact of ZIKV impact on fetuses [60, 64]. ZIKV infection of NHPs, specifically rhesus macaques, appears to closely resemble the viral kinetics and immune response found in humans [18, 58, 59, 61]. However, no animal model is a perfect substitute for an actual human infection, as disease characteristics such as rash and fever are often not always associated with the NHP and mouse models [58, 59, 61].

We here present the first human study of the ontogeny of the immune responses against ZIKV. We demonstrate that the virus is no longer detectable from the patient’s plasma by D15 POS. This matches the viral kinetics seen in NHP infections which generally have undetectable virus in the plasma by D7 post-infection [18, 58, 59, 61]. This seems appropriate given the suspected 3–14 day incubation period in mosquito-borne transmission in humans [65]. This short detection window of viral nucleic acid in plasma underlines the difficult reality physicians will face when attempting to diagnose ZIKV at the end of an acute infection and the need for a post-acute diagnostic test [66]. Previously, it was thought that ZIKV was more readily detectable in the saliva than in plasma, but the window for detection, on average, remained the same [67]. Moreover, it was found that saliva alone was insufficient for diagnosis as replicate saliva samples varied greatly in virus titer when compared to the consistency of being able to detect ZIKV in plasma [67]. Only recently has it been shown that ZIKV persists in whole blood substantially longer than in plasma, which may allow for more sensitive testing and a greater window of diagnosis [68]. Furthermore, it is unclear as to what ZIKV persistence in whole blood may mean for the potential safety of whole blood and blood products from potential asymptomatic donors that were in ZIKV endemic areas. It has, however, been shown that these blood transmission events were rare in French Polynesia [69].

The Pb response in Hu0015 appears to match the kinetics of previously published Pb studies in ZIKV-infected NHPs; elevated at D5 and peaking at D7 post-infection, before rapidly contracting [59]. Furthermore, the kinetics of the Pb response in a human primary ZIKV infection appear to occur at a similar rate when compared to that of human primary DENV Pb responses [49, 50, 54, 55]. This information may be useful for the isolation of ZIKV-specific monoclonal antibodies, since the ability to clone antibodies from this acute Pb population has led to the isolation of several potent monoclonal antibodies directed against ZIKV which may be pivotal in ZIKV diagnostics and therapies [17, 55, 70, 71].

Some of the specificities of the antibodies generated in this patient were unexpected. Cross-reactive DENV binding antibodies were observed soon after infection in Hu0015’s plasma. However, these antibodies did not appear to neutralize DENV. While Stetter *et*. *al*. [17] have shown that some of the mAbs isolated from acute ZIKV infection (without prior DENV exposure) can neutralize DENV, we have found distinctly the opposite result in the plasma from Hu0015 at all time points tested. It is possible, therefore, that while some DENV neutralizing antibodies may be present at low frequencies, the bulk of the antibodies in the plasma of our ZIKV-infected patient do not neutralize DENV.

While the concept of ADE in DENV infections has been well characterized, less is known as to how ZIKV seropositive status might contribute to enhancement of DENV or ZIKV infection [17, 51, 53]. ADE was first described by Halstead in 1973 in which he performed a series of *in vitro* and *in vivo* experiments addressing the issue of enhancement of DENV infection [72–75]. Interestingly, Hu0015’s plasma did not enhance DENV infection *in vitro*, despite the presence of DENV binding antibodies. Perhaps this lack of DENV ADE is related to the lower levels of DENV-cross-reactive antibodies which did not reach a sufficient threshold to induce ADE in our patient. By contrast, ADE of ZIKV infection was present at D48 POS, when ZIKV IgG antibodies were at higher concentrations than they were during the acute phase (Fig 8).

The ontogeny of the T-cell response against ZIKV has yet to be carefully defined in humans, although there are some animal models that may be instructive [76]. The Hu0015 ZIKV-specific CD8^+^ T-cell immune response is one of the first to be analyzed using a ZIKV-specific MHC class I tetramer. It has previously been shown, that the CD8^+^ T-cell responses against DENV primarily target the non-structural proteins [47, 77]. Surprisingly, in Hu0015, the T-cell response against primary ZIKV infection was directed entirely against ZIKV-E. New results clearly show that the CD8^+^ T-cell response in predominately against ZIKV-E in several other cases of primary ZIKV infection in humans, thus further differentiating the CD8^+^ T-cell response from DENV infection [78]. Previous CD8^+^ T-cell YF-specific MHC class I tetramer studies have shown that the T-cell response peaks at D30 post-vaccination [79]. With the case of ZIKV infection in Hu0015, it is interesting to note that the peak of tetramer positive CD8^+^ T-cells occurred at D21 POS. The most likely explanation for the difference is the estimated incubation period of 3–14 days in which the virus is replicating in the patient before the onset of symptoms [65]. Another explanation may also be that the previously mentioned YF vaccination study only measured D14 and D30 post-vaccination and the peak may have occurred earlier than D30 post-vaccination. Additionally, the CD8^+^ tetramer response after DENV infection shows remarkably similar peaks between D7 to D14 POS [80–82].

In conclusion, the ZIKV outbreak in Miami, FL in 2016 highlighted that ZIKV can pose a severe threat to public health in the US and may become a re-occurring reality. Importantly, the degree to which our results from this single DENV-naïve individual can be extrapolated to the entire DENV-naïve population is unknown. However, a longitudinal analysis of the B- and T-cell ontogeny of a large cohort will require considerable resources. Here, our detailed longitudinal analysis provides the groundwork for larger studies in the future. We followed the evolution of the B- and T-cell response against ZIKV and saw peak immune responses between D15 and D21 POS (Fig 8). The highest neutralization activity in plasma occurred during the peak of the ZIKV-specific IgM and IgG antibody response. We detected a rapid Pb expansion and described the ontogeny of the generation of cross-reactive antibodies against DENV after primary ZIKV infection. We observed ADE of ZIKV with Hu0015’s plasma at D48 POS, but there was no ADE of DENV2 infection despite the presence of cross-reactive antibodies against DENV. For the first time, we have described the ontogeny of the plasmablast, antibody, and T-cell immune responses in one of the earliest locally acquired ZIKV infections in the contiguous US.

## Supporting information

S1 Fig Tetramer positive cells overlaid on CD8^+^ T-cells by memory phenotype. The tetramer positive CD8^+^ T-cells over time, overlaid on top of the naïve, effector memory, central memory, and effector memory CD8^+^ T-cells based on expression of CCR7 and CD45RA markers.
(TIF)

S2 Fig Phenotype of CD8^+^ T-cells over time. An in-depth characterization of the phenotype of CD8^+^ T-cells was performed by ICS.
(TIF)

S3 Fig IFN-γ expressing CD8^+^ T-cells overlaid on CD8^+^ T-cells. An ICS of the CD8^+^ IFN-γ^+^ T-cells at D21 POS revealed an overexpression of cytotoxic markers PD1 and Granzyme B, and a downregulation of CX3CR1.
(TIF)

S4 Fig Flow cytometry controls for ZIKV-naïve and ZIKV-exposed PBMCs. Controls for the T-cell flow cytometry experiments.
(TIF)

S1 Table Antibody clones used in flow cytometry. Antibodies used in the ICS, tetramer, and plasmablast stainings.
(TIF)
