# Supplementary figures and images for: Ontogeny of the B- and T-cell response in a primary Zika virus infection of a dengue-naïve individual during the 2016 outbreak in Miami, FL
Source: PLoS Negl Trop Dis. 2017 Dec 21;11(12):e0006000. doi: 10.1371/journal.pntd.0006000 (PMC5755934; doi:10.1371/journal.pntd.0006000)

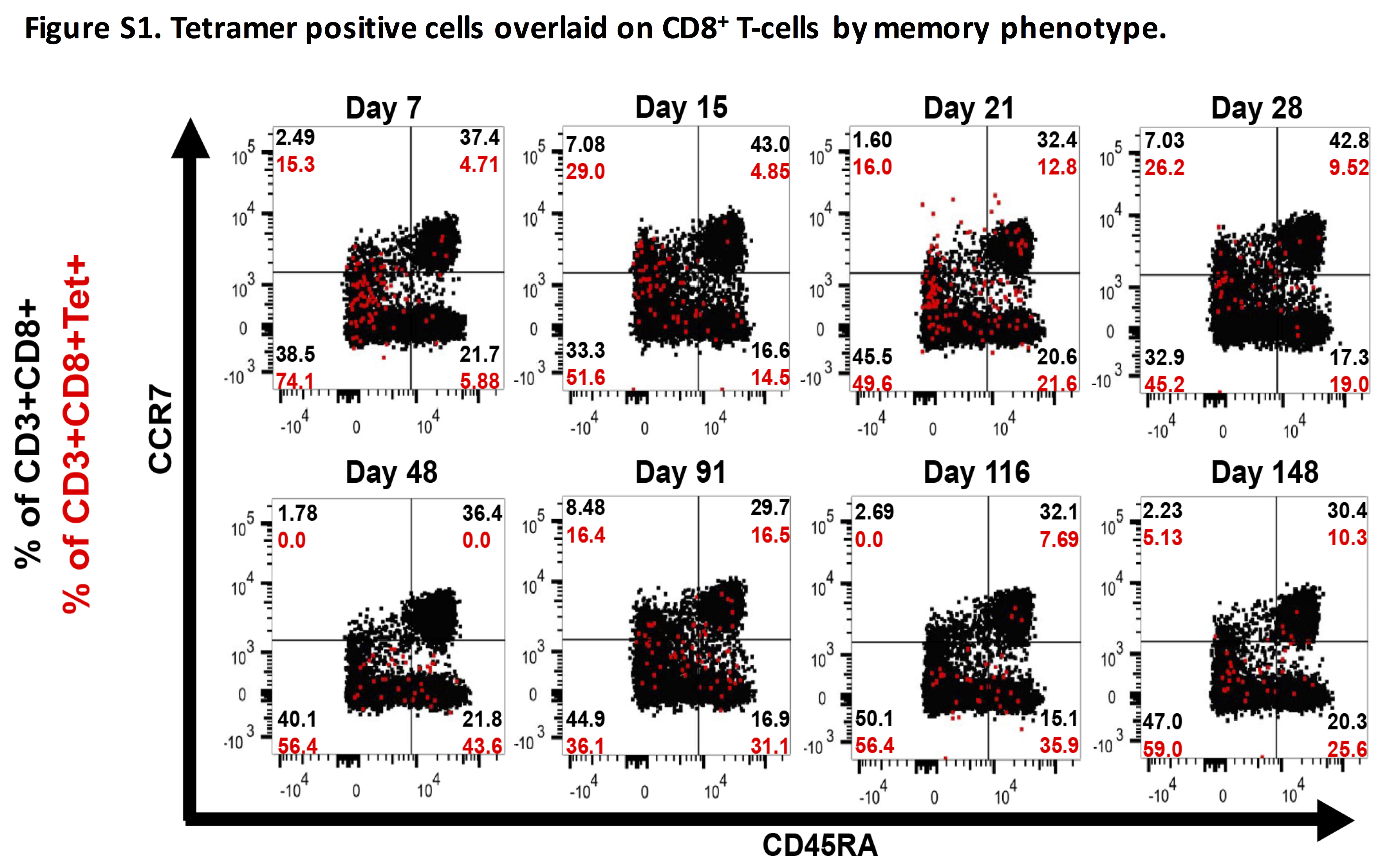

Supplement: S1 Fig — The tetramer positive CD8+ T-cells over time, overlaid on top of the naïve, effector memory, central memory, and effector memory CD8+ T-cells based on expression of CCR7 and CD45RA markers. (TIF) [file pntd.0006000.s001.tif]

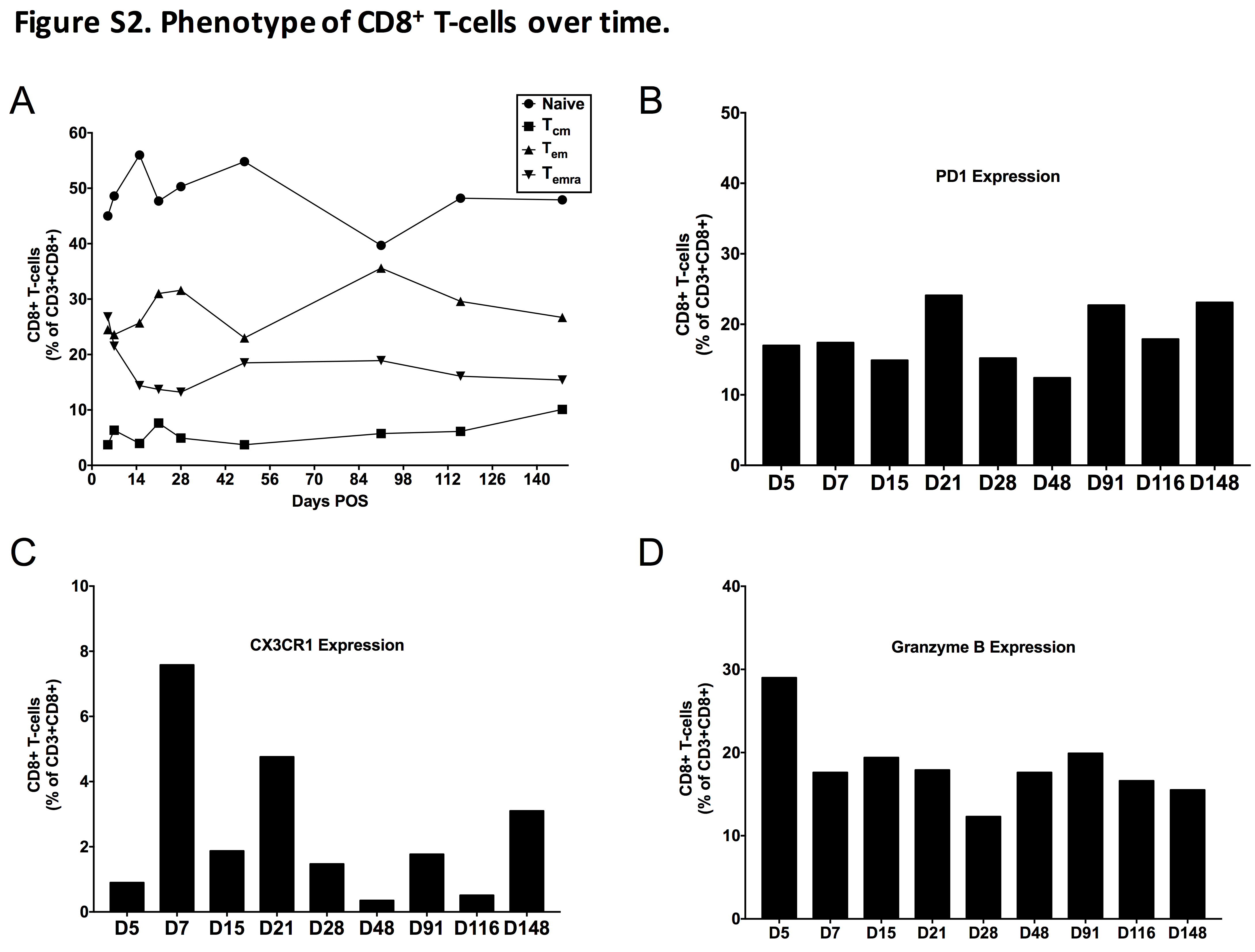

Supplement: S2 Fig — An in-depth characterization of the phenotype of CD8+ T-cells was performed by ICS. (TIF) [file pntd.0006000.s002.tif]

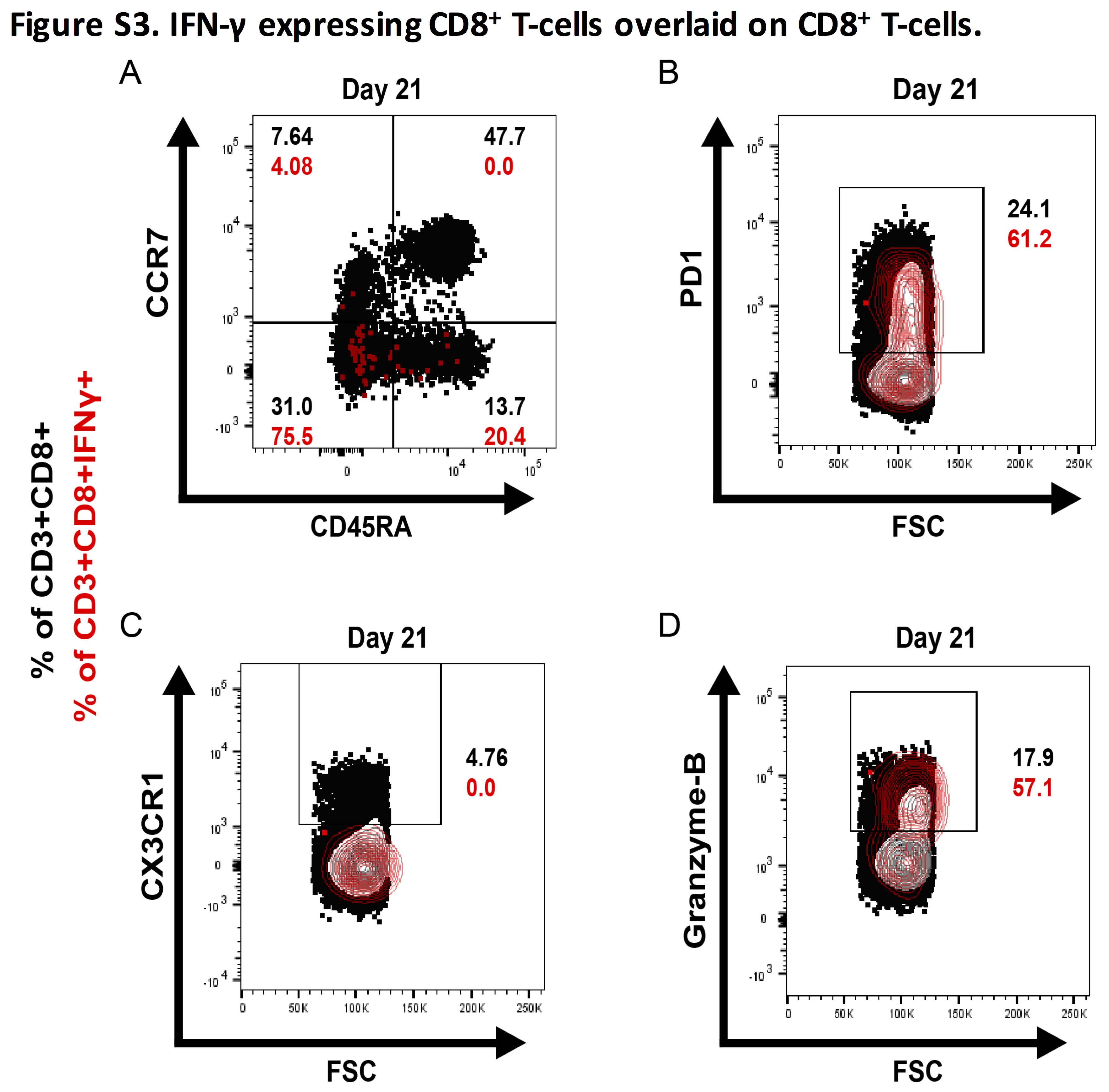

Supplement: S3 Fig — An ICS of the CD8+ IFN-γ+ T-cells at D21 POS revealed an overexpression of cytotoxic markers PD1 and Granzyme B, and a downregulation of CX3CR1. (TIF) [file pntd.0006000.s003.tif]

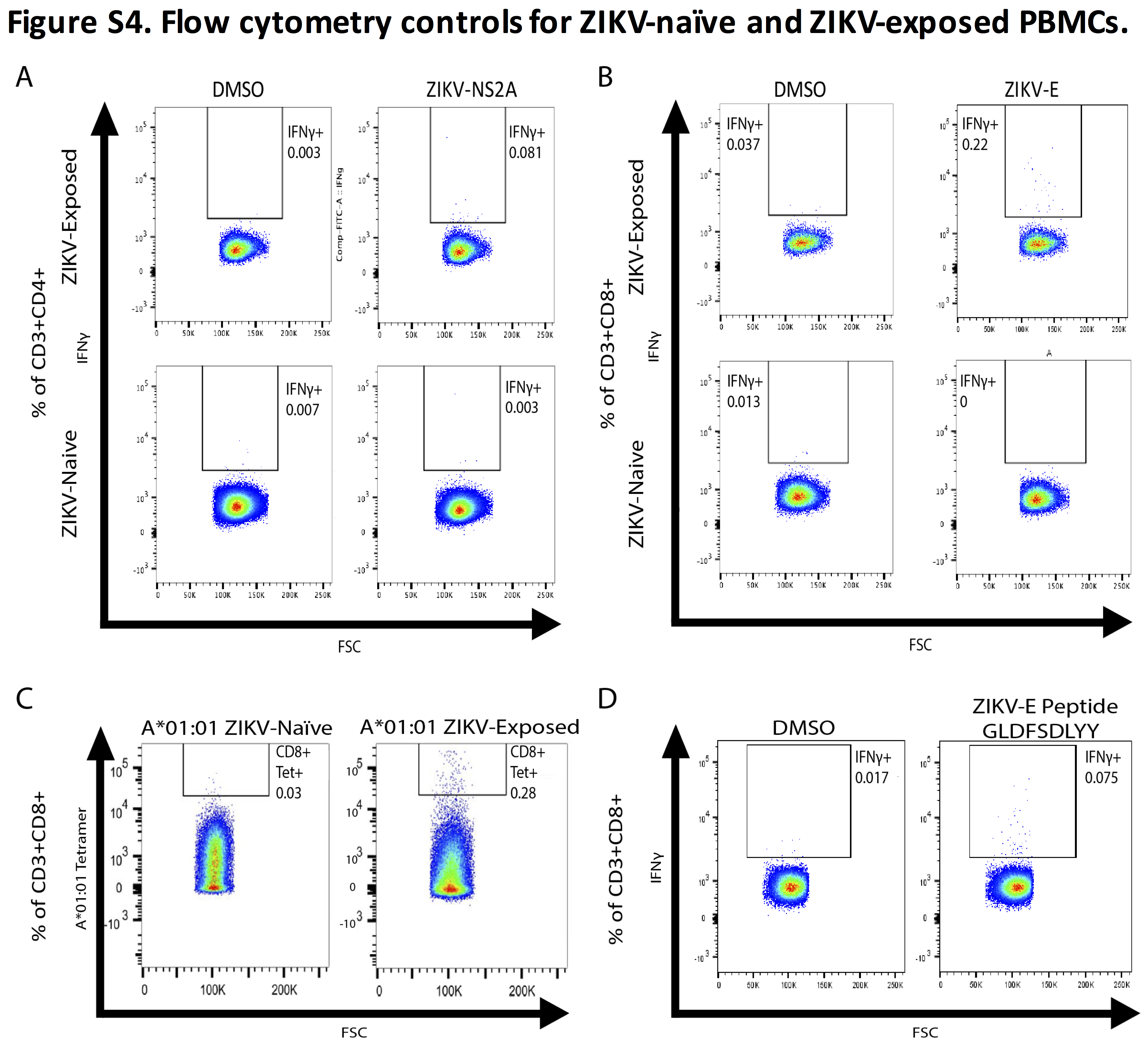

Supplement: S4 Fig — Controls for the T-cell flow cytometry experiments. (TIF) [file pntd.0006000.s004.tif]

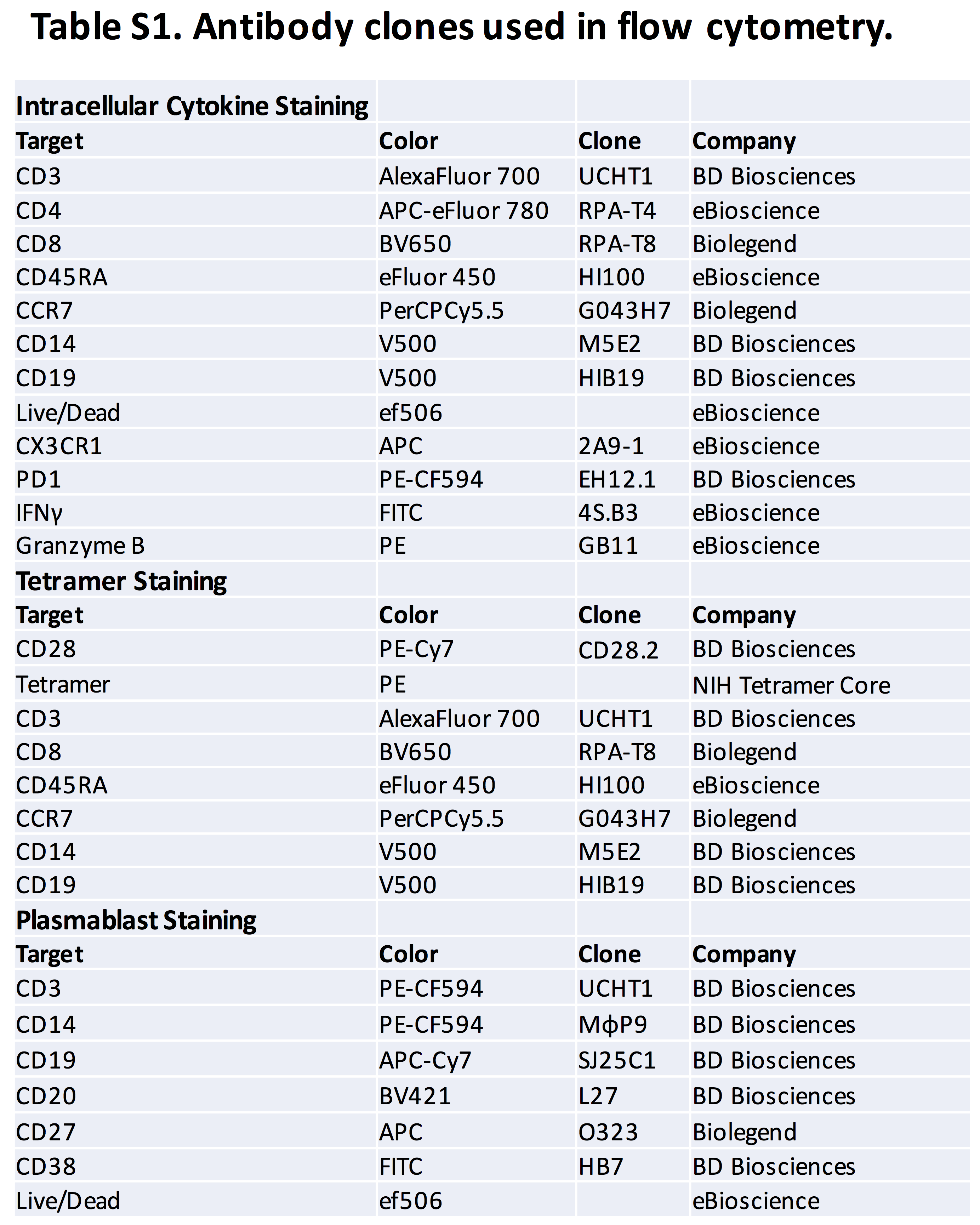

Supplement: S1 Table — Antibodies used in the ICS, tetramer, and plasmablast stainings. (TIF) [file pntd.0006000.s005.tif]
